# In This Issue

**Published:** 2000

## Introduction

The use of animals to model humans has long been an integral part of medical and scientific research into human functions and conditions. The development of animal models for alcoholism began in the 1940s. Since that time, a variety of animals have been used to model different drinking behaviors and to study how alcohol damages different bodily organs. Animal models also have helped scientists to analyze the changes in brain chemistry that occur when alcohol is consumed. Perhaps most promising, genetically altered animal models are proving to be invaluable in the search for genes that may be involved in the development of alcoholism. Indeed, a key advantage of animal research, especially research into complex disorders such as alcoholism, is that it enables scientists to simplify complex behaviors by producing fundamental models that are relevant to the human situation.

In developing our issue of *Alcohol Research & Health* on “Animal Models,” we quickly found that the existing literature on this topic far surpassed our page limit for a single issue. To include every topic, only a cursory mention would be possible. Though it breaks with tradition, we felt it would serve our readers better to cover each topic in depth and to dedicate two full issues to the study of animal models in alcohol research.

The first issue gave a broad perspective on the use of animals for investigating the behavioral and physiological effects of alcohol, with only a brief mention of the burgeoning field of genetics research. This second issue examines in greater detail the use of animals in the search for the genes involved in alcoholism. Together these two issues will provide an excellent review of how animals are helping scientists to better understand the complexities of alcoholism and the effects of alcohol on the human mind and body.

Enoch Gordis, M.D., Director

National Institute on

Alcohol Abuse and Alcoholism

National Institutes of Health

## MODELS OF PSYCHIATRIC DISORDERS AND THEIR RELEVANCE TO ALCOHOLISM

Animal models have been developed to study a number of disorders, including alcoholism, and have also been used to study the interactions between alcoholism and co-occurring psychiatric disorders. Those models allow researchers studying psychiatric disorders to test specific hypotheses under highly controlled conditions, using methods that are either impossible or unethical to use in humans. Dr. Robert Hitzemann describes animal models of schizophrenia, depression, fear and anxiety, and alcoholism, with a special emphasis on their use in the search for genes that contribute to the disorders. He also discusses the various criteria used to evaluate animal models and reports on the use of animal models in the study of the relationship between alcoholism and co-occurring psychiatric disorders. Such models may prove useful in determining how best to treat patients with alcoholism and a co-occurring disorder. (pp. 149–158)

## SELECTED LINES AND INBRED STRAINS

Rodent strains that are generated using various breeding techniques and which exhibit certain alcohol-related traits are a mainstay of research investigating the genetic basis of alcoholism. In one approach, animals that show a high or low incidence of a certain trait are repeatedly mated to increase the strength of that particular trait. Alternatively, researchers can mate male and female siblings, regardless of any particular trait, over several generations to create an inbred strain in which all animals have the same genetic makeup. Then several such inbred strains can be compared with respect to alcohol-related traits in order to identify correlations between those traits and certain genetic characteristics. Dr. Nicholas J. Grahame reviews the techniques used to produce selected lines and inbred strains and discusses the advantages and limitations of these animal models. (pp. 159–163)

## THE CANDIDATE GENE APPROACH

Based on the results of various genetic approaches, researchers can identify specific genes that may contribute to a particular disorder, such as alcoholism. The role of those genes can then be assessed directly using the candidate gene approach, which explores the association of certain gene variants with the presence or absence of an alcohol-related trait. Such analyses, which are commonly conducted in humans but can also be used in animal models, can identify genes or gene variants that have only a relatively small influence on the trait under investigation, report Drs. Jennifer M. Kwon and Alison M. Goate. To use this approach successfully, however, it is imperative that researchers already have some understanding of the biological mechanisms underlying the disorder under investigation. (pp. 164–168)

## QUANTITATIVE TRAIT LOCUS ANALYSIS

Like other complex behaviors or characteristics, alcoholism is a quantitative trait—that is, it varies continuously across a population and is determined by numerous genetic and environmental factors. DNA regions mediating those influences are called quantitative trait loci (QTLs), and researchers are now beginning to determine the location (i.e., map) of those QTLs. In their efforts to identify alcoholism-related QTLs, researchers rely primarily on animal models. Dr. Judith E. Grisel presents examples of QTL analysis in animal research. This technique has resulted in the identification of several DNA regions that may contribute to certain alcohol-related traits, such as withdrawal susceptibility. (pp. 169–174)

## TRANSGENIC AND KNOCKOUT MICE IN ALCOHOL RESEARCH

How do specific genes contribute to alcoholism risk? Researchers studying the roles of particular genes in alcohol-related behaviors and alcoholism are making progress thanks to advances in genetic engineering. Dr. Barbara J. Bowers describes the creation and use of transgenic mice, in which a foreign gene is integrated into an animal’s genetic material, and knockout/knock-in mice, in which targeted genes are rendered either nonfunctional or are altered. Both of these animal models are currently being used in alcohol research to determine how genes may influence the development of alcoholism in humans. (pp. 175–184)

## GENETICS OF ALCOHOL-INDUCED BEHAVIORS IN *DROSOPHILA*

Although researchers most commonly use rodents as animal models of alcoholism, some studies also have been conducted in the fruitfly *Drosophila melanogaster*—a model that is particularly useful for studying the genetic basis of mammalian development and behavior. As Dr. Ulrike Heberlein reports, investigators have begun to measure alcohol sensitivity in *Drosophila* and to identify genetic changes (i.e., mutations) associated with increased or decreased sensitivity. These investigations have pinpointed several mutations in a cell-signaling system called the cAMP system, which plays a role in many cellular regulatory processes. This signaling system also is affected in human alcoholics, supporting the validity of *Drosophila* as a model system of human alcoholism. (pp. 185–188)

## FUTURE DIRECTIONS OF ANIMAL MODELS IN ALCOHOL RESEARCH

Not only does a person’s genetic makeup help determine his or her risk for alcoholism, alcohol consumption, itself, can alter the expression of certain genes. Whether such genes are involved in the development of alcoholism or are products of this disease, however, is still unknown. Drs. P. J. Brooks and Robert H. Lipsky examine promising new ways these questions can be answered. Two techniques, differential expression and the automated gene expression analysis, already have produced significant results. Differential expression, for example, has determined that a key area in the brain (i.e., the nucleus accumbens) is involved in the development of alcoholism, whereas automated gene expression analysis has brought scientists much closer to one day knowing for certain each and every gene involved in alcohol-related behavior. A third technique, the use of viral vectors, or man-made viruses, developed to express specific genes, are being used to determine whether the genes identified above produce alcoholism or are products of this disease. (pp. 189–192)

